# Physical Activity Is Associated with Improved Visuomotor Processing in Older Adults with Moderate and Advanced Glaucomatous Visual Field Defect: A Cross-Sectional Study

**DOI:** 10.3390/ijerph19031760

**Published:** 2022-02-03

**Authors:** Teresa Zwierko, Wojciech Jedziniak, Beata Florkiewicz, Piotr Lesiakowski, Marta Śliwiak, Marta Kirkiewicz, Wojciech Lubiński

**Affiliations:** 1 Laboratory of Kinesiology, Functional and Structural Human Research Center, Institute of Physical Culture Sciences, University of Szczecin, 70-240 Szczecin, Poland; wojciech.jedziniak@usz.edu.pl (W.J.); beata.florkiewicz@usz.edu.pl (B.F.); 2 Department of Physical Education and Sport, Pomeranian Medical University, 70-123 Szczecin, Poland; lesiakowskipiotr@gmail.com; 3 II Department of Ophthalmology, Pomeranian Medical University, 70-111 Szczecin, Poland; mkierznowska@gmail.com (M.Ś.); martakiszkielis@gmail.com (M.K.); lubinski@pro.onet.pl (W.L.)

**Keywords:** glaucoma, reaction time, physical activity

## Abstract

Glaucoma affects a wide spectrum of daily essential activities in older adults. This study examined whether older adults with moderate and advanced stages of glaucoma exhibit differences in visuomotor task performance compared with age- and gender-matched ophthalmologically healthy control subjects and estimated the effects of physical activity (PA) levels, age, and severity of visual impairment on patients’ visuomotor task performance. Sixty older adults with moderate glaucoma, advanced glaucoma, and normal sight participated in the study. Visuomotor processing was assessed using laboratory-based simple and complex visuomotor reaction tasks. Monocular Humphrey Visual Field and binocular Humphrey Esterman Visual Field tests were used to estimate visual field defect severity. The International Physical Activity Questionnaire was used to assess PA levels. Participants with glaucoma had poorer scores in visuomotor tasks compared to participants with normal sight. Glaucoma patients’ PA levels, age, and binocular visual field defect explained 54% of the variation in complex reaction time. Low PA levels were identified as a risk factor for visuomotor processing decline. Compensatory mechanisms to improve the efficiency of visual field scanning in patients with more severe visual field defects may exist.

## 1. Introduction

Ageing leads to a progressive impairment in the ability of older adults to detect, discriminate, integrate, and respond to visual and verbal information, with accelerated impairment in those affected by neurodegenerative lesions [1,2]. This accelerated impairment is also observed in older adults with age-related eye disease [3,4,5].

Glaucoma is a an ophthalmological disease characterized by slow degeneration of retinal ganglion cells resulting in the deterioration of visual function [6]. Glaucoma is the first cause of irreversible blindness worldwide; it is predicted that the number of adults with glaucoma will reach 112 million by 2040 [7]. Glaucomatous visual field loss may significantly affect functional daily living activities, in particular activities with heavy demands on visuomotor processing. Previous studies have found that glaucoma negatively affects gait and mobility [8,9,10], eye-hand coordination [11,12], and driving ability [13,14,15]. An increased risk of motor vehicle collisions has also been observed in glaucoma patients [16,17,18]. However, other studies have found that the extent of the visual field defect is of minor importance regarding functional performance in glaucoma patients during a limited duration of driving or using driving simulators, as they compensate for the visual field deficit by adapting their visual scanning behaviors [19,20].

It appears that, as tasks become more demanding, glaucoma patients’ performance may deteriorate. The present study seeks to address this issue by investigating visuomotor reaction time with increasing difficulty in laboratory conditions. Laboratory-based tasks allow for the influence of task-specific expertise when undertaking “real life” tasks such as driving to be controlled. Furthermore, it is important to know the level of glaucomatous visual field defect severity that causes visuomotor processing decline.

Physical activity (PA) has been widely reported as important for maintaining and improving brain health and reducing age-related cognitive decline [21,22,23]. Although it is generally accepted that PA can enhance cognitive function, most studies [23,24] have been in healthy older adults; thus, the applicability of these findings to older adults with glaucoma is debatable. It is known that some kind of PA can reduce the development and progression of glaucoma by lowering intraocular pressure (IOP) and increasing ocular blood flow [25,26,27,28]. However, little is known about how PA, particularly for patients with moderate and advanced stage glaucoma, affects visuomotor performance. Specifically, to our knowledge, the assessment of the relationship between daily PA and simple and complex visuomotor task performance in older adults with moderate and advanced glaucomatous visual field defect has not yet been evaluated.

Thus, the purpose of the present study is to examine how daily PA, age, and severity of visual impairment affects simple and complex visuomotor processing in patients with glaucoma. In accordance with previous findings [11,14,15], patients with glaucoma are hypothesized to achieve poorer scores in visuomotor processing tasks relative to those with no eye disease. Furthermore, in line with earlier studies [29,30,31,32], it is expected that severe visual field defects and lower PA levels will be associated with larger deficits in the executive functioning (as indicated by visuomotor task performance) of patients with glaucoma. This study may help to establish significant predictors of performance in laboratory-based visuomotor tasks.

## 2. Materials and Methods

### 2.1. Participants

Using the European Glaucoma Society classification, 20 older adults (aged 65.30 ± 7.50 years) with moderate stage glaucoma (MG) in at least one eye (between −6 dB and −12 dB) and 20 older adults (aged 66.35 ± 6.35 years) with advanced stage glaucoma (AG) in at least one eye (worse than −12 dB) were recruited. A control group (CG) comprised of 20 age- and gender-related ophthalmologically healthy subjects. The sample dimension analysis was performed using G*Power 3.1 software (Heinrich Heine Universität Düsseldorf, Düsseldorf, Germany) [33]. Based on a priori analysis for the one-way ANOVA method, we adopted a power of 0.80, an alpha of 0.05, and an effect size of 0.42 (medium to large effect size).

**Figure 1:**
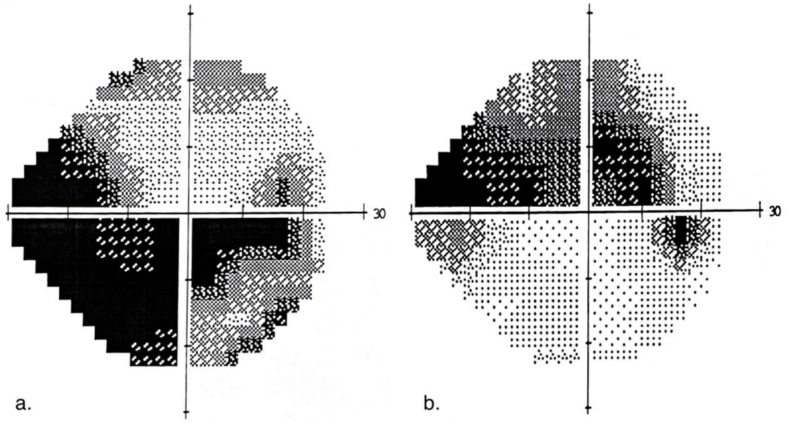
Example of visual field defect: (**a**) with advanced stage of glaucoma MD of −20.42 dB (**b**) with moderate stage of glaucoma MD of −10.09 dB.

The following exclusion criteria were used: (1) glaucoma with unstable IOP; (2) systemic diseases with known effects on retinal function (e.g., diabetes); (3) other ocular diseases (e.g., cataract); (4) neurological disease; (5) severe cardiovascular diseases; and (6) moderate cognitive impairment as determined by the Mini-Mental State Examination (MMSE score < 26) [34]. Research groups were comparable in terms of underlying health conditions, aside from glaucoma. Demographic and ophthalmological characteristics of participants are presented in Table 1. All participants underwent an ocular examination that included: best-corrected visual acuity (BCVA) assessment using a Snellen chart, perimetry (Humphrey Field Analyzer, Crl Zeiss Meditec, Inc., Dublin, CA, USA), and static perimetry 24–2 (white on white) Swedish Interactive Threshold Algorithm (SITA) standard visual field testing. Mean deviation (MD) scores were used to assess the severity of visual field loss for each eye. The binocular Humphrey Esterman Visual Field generated a binocular visual field score. A grid of 120 test points with light intensity of 10 dB was used to examine more than 130° of the visual field. Binocular visual field defects (VFDS) with a number of omitted points and Esterman coefficients were analyzed. MD scores were used to assess the severity of visual field loss for each eye. Each eye was classified as either the “better” or “worse” eye based on the MD score. Examples of glaucomatous visual field defects for advanced and moderate stages of glaucoma are presented in Figure 1.

The study was conducted in accordance with the Declaration of Helsinki and approved by the local bioethical committee (No. 10/KB/VI/2017). Before examination, subjects were informed about the testing protocol. All participants signed a written informed consent and were permitted to withdraw from the study at any time.

### 2.2. Visuomotor Processing Evaluation

Visuomotor processing was evaluated using a test battery from the Vienna Test System (Dr Schuhfried Medizintechnik GmbH, Vienna, Austria). The simple reaction time test for visual stimuli in version S1 was applied. In this task, 28 light stimuli (yellow light) are randomly presented to participants over a time period of 2.5–6.0 s. To evaluate eye-hand reaction time, participants were required to respond to the stimuli by releasing a “waiting button” and pressing a “response button”. The main variables calculated were: reaction time—the period of time between the appearance of the stimulus and the start of movement to release the “waiting button” (ms); motor time—the period of time between releasing the “waiting button” and pressing the “response button” (ms); and total reaction time (as indicated by reaction time + motor time). All reactions were required to be accurate (i.e., participants must have released the “waiting button” and pressed the “response button” after the stimulus had been presented).

**Figure 2:**
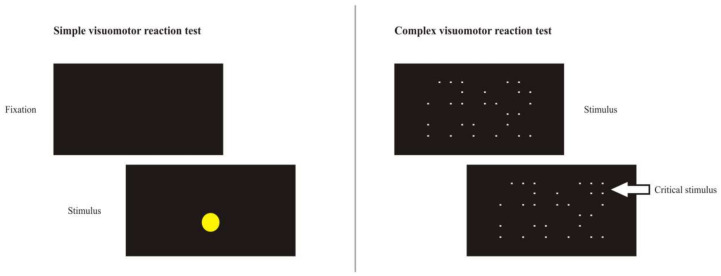
Schematic representation of the stimuli presented in the simple and complex visuomotor reaction tasks.

The Signal Detection Test evaluated the eye-hand reaction time in a complex visuomotor task. The test measured the visuospatial differentiation of a relevant signal within irrelevant signals. The standard S1 version with white signals (dots) on a black background was used. Dots were displayed over the entire screen area (24 inch) with the dots appearing and disappearing pseudo-randomly. Participants were required to respond with a keypress when a constellation of four dots forming a square was presented (i.e., the critical stimulus) and to refrain from responding when other constellations (i.e., distractor stimuli) were presented. Participants had 3.75 s to detect and respond to the critical stimulus. The test was subdivided into 20 partial intervals. Each partial interval consists of 50 steps (dot changes) with three critical stimuli presented per partial interval. The main variables calculated were the number of correct responses to critical stimuli and the number of critical stimuli not responded to (i.e., missed responses), with the median reaction time (ms) to make a correct response over the course of the entire test used as an indicator of visuomotor processing speed. Schematic representations of the stimuli presented in the simple and complex visuomotor reaction tasks are presented in Figure 2.

### 2.3. Physical Activity (PA) Measure

The long version of the International Physical Activity Questionnaire (IPAQ) was used to measure self-reported participation in PA [35]. The IPAQ included questions on frequency (days/week) and duration (min/day) of vigorous and moderate intensity PA and walking during the previous 7 days. PA was classified into four domains (work, transport, household and leisure) within the categories of walking, moderate-intensity PA, and vigorous-intensity PA. PA metabolic equivalent (MET, min/week) was calculated according to the following formula: number of days spent doing the activity × average duration of the activity per day × MET coefficient value. The MET coefficient values were as follows: walking = 3.3, moderate activity = 4, vigorous activity = 8, cycling as a form of transport = 6, intensive activity near the house = 5.5. PA in total MET minutes of PA per week was then calculated as the sum of the MET minutes obtained in each category.

### 2.4. Statistical Analysis

The Shapiro–Wilk test was used to verify the normal Gaussian distribution of the data, and Levene’s test was used to check the homogeneity of variance. If the data were normally distributed within groups, a one-way ANOVA was used to analyze the effect of the group on the dependent variables. Effect size was analyzed using partial eta-squared (ηp^2^). Post hoc tests were performed using a Bonferroni correction; a *p* value < 0.05 was considered significant. The magnitude of effect sizes for pairwise comparisons was determined using Cohen’s *d*. Effect sizes obtained using Cohen’s *d* were characterized as small (0.2), medium (0.5), or large (0.8) [36]. If the data were not normally distributed, the Kruskal–Wallis test was used to analyze the effect of the group on the dependent variables, with Dunn’s multiple comparison tests applied as post hoc analyses. For glaucoma patients, Pearson’s correlation coefficient (*r*) was used to assess the relationship between visuomotor task performance and visual field defect severity, visuomotor task performance and age, and visuomotor task performance and PA. Backward stepwise multiple regression analysis was performed to assess the influence of independent variables on simple and complex reaction time. All calculations were performed with STATISTICA ver. 13.3 (TIBCO Software Inc., Palo Alto, CA, USA).

## 3. Results

The simple and complex visuomotor test results for the glaucoma patient groups and the control group are presented in Table 2. No significant effect of the group on simple reaction time was observed (*F*_(2,56)_ = 2.261, *p* = 0.113, ηp^2^ = 0.074). However, a significant effect of the group on simple motor time was found (*F*_(2,56)_ = 6.288, *p* = 0.003, ηp^2^ = 0.183). Specifically, compared to the CG, AG patients demonstrated poorer simple motor time results (268.15 ± 85.11 ms vs. 191.60 ± 65.40 ms, *p* = 0.004, *d* = 1.008). Similarly, MG patients took longer than the CG (248.65 ± 61.23 ms vs. 191.60 ± 65.40 ms, *p* = 0.038, *d* = 0.901). Simple motor time did not differentiate between patients with MG and AG (268.15 ± 85.11 ms vs. 248.65 ± 61.23 ms, *p* > 0.05, *d* = 0.263).

There was a significant effect of group on total reaction time (*F*_(2,56)_ = 7.275, *p* = 0.002, ηp^2^ = 0.203). Significant differences between AG patients and the CG were observed (598.45 ± 106.73 ms vs. 481.30 ± 105.85 ms, *p* = 0.002, *d* = 1.102), as were differences between MG patients and the CG (572.15 ± 92.56 ms vs. 481.30 ± 105.85 ms, *p* = 0.020, *d* = 0.910). In the complex visuomotor reaction task there was a significant effect of group on correct reactions (*F*_(2,56)_ = 6.716, *p* = 0.002, ηp^2^ = 0.193). The CG had better results than both the AG patients (50.05 ± 4.39 vs. 43.65 ± 6.36, *p* = 0.003, *d* = 1.171) and the MG patients (50.05 ± 4.39 vs. 44.75 ± 6.59, *p* = 0.018, *d* = 0.947). However, there was no significant difference between patients with AG and patients with MG (44.75 ± 6.59 vs. 43.65 ± 6.36, *p* > 0.05, *d* = 0.170).

A significant effect of group on the number of missed responses was observed (*F*_(2,56)_ = 5.515, *p* = 0.001, ηp^2^ = 0.165). Patients with AG had worse results than CG (14.55 ± 6.16 vs. 8.90 ± 4.81, *p* = 0.009, *d* = 1.022), as did patients with MG (13.60 ± 6.31 vs. 8.90 ± 4.81, *p* = 0.036, *d* = 0.838).

The most noticeable effect of group was observed in relation to the complex reaction time (*F*_(2,56)_ = 14.427, *p* < 0.001, ηp^2^ = 0.340). The AG group had worse results than the CG (1624.33 ± 257.20 ms vs. 1210.20 ± 241.26 ms, *p* < 0.0001, *d* = 1.661) but similar results to the MG group (1624.33 ± 257.20 ms vs. 1502.35 ± 275.52 ms, *p* = 0.269, *d* = 0.458). The CG also demonstrated better performance than the MG group (1502.35 ± 275.52 ms vs. 1210.20 ± 241.26 ms, *p* = 0.002, *d* = 1.128). For all performance parameters of the visuomotor reaction tests, no significant differences between the MG patients and AG patients were observed.

Table 3 shows the results of the glaucoma patient correlation analysis between visuomotor reaction task performance and VF defect severity, visuomotor reaction task performance and age, and visuomotor reaction task performance and PA. Significant correlations were observed between age and visuomotor reaction task performance with associations between age and simple motor time (*r* = 0.396, *p* < 0.05), age and total reaction time (*r* = 0.370, *p* < 0.05), age and correct reactions (*r* = −0.389, *p* < 0.05), age and missed reactions (*r* = 0.335, *p* < 0.05), and age and complex reaction time (*r* = 0.650, *p* < 0.01). Visual field defect scores positively correlated with simple motor time (*r* = 0.433, *p* < 0.05), total reaction time (*r* = 0.47, *p* < 0.05), and complex reaction time (*r* = 0.409, *p* < 0.05). Higher energy expenditures related to daily PA correlated with simple motor time (*r* = −0.330, *p* < 0.05), total reaction time (*r* = −0.389, *p* < 0.05), correct reactions (*r* = 0.341, *p* < 0.05) and complex reaction time (*r* = −0.659, *p* < 0.01).

**Figure 3:**
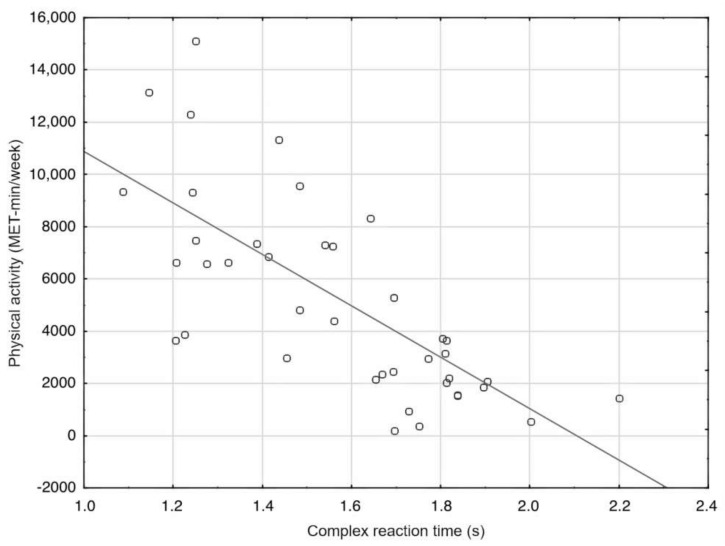
PA as a main predictor for complex reaction time in glaucoma patients, y = 20,725.18 − 9841.17x; *R^2^* = 0.434.

A backward stepwise multiple regression model using baseline age, BCVA, MD, VFDS, and PA as predictors showed that only VFDS (*ß* = 0.39, *p* = 0.009) and PA (*ß* = 0.28, *p* = 0.05) were independently associated with simple reaction time (adjusted R^2^ = 0.258, *F*_(2,37)_ = 7.782, *p* < 0.002). However, the regression model composed of PA (*ß* = −0.41, *p* = 0.003), age (*ß* = 0.37, *p* = 0.008), and VFDS (*ß* = 0.18, *p* = 0.121) explained a higher percentage of variation in complex reaction time (adjusted *R^2^* = 0.54, *F*_(3,36)_ = 16.803, *p* < 0.0001). PA accounted for 43% (*R^2^* = 0.434) of the variance in complex reaction time in glaucoma patients (Figure 3).

## 4. Discussion

The findings of this study suggest that the glaucoma patients performed poorly in the visuomotor processing tasks compared to participants with no eye disease, particularly in the complex visuomotor reaction task. In the simple visuomotor reaction task, simple reaction time did not differ between the glaucoma and control groups; however, there were significant differences in simple motor time between each of the glaucoma groups and the control group. Motor time in this task referred to the time elapsed between releasing the “waiting button” and pressing the “response button” following the stimulus appearance, with less time indicating greater hand movement speed. Task efficiency in this case depended on coordination of eye and hand movements. Previous studies have shown eye-hand coordination impairment in glaucoma patients. For instance, Kotecha et al. [12] analyzed the reach-to-grasp behavior of glaucoma patients compared to normal-sighted controls and observed delays in average movement onset (mean delay = 100 ms) and overall movement time (mean delay = 140 ms) in the glaucoma patients. Planning and initiation of reaching-to-objects during reaching-and-grasping tasks appeared to account for the movement delays. The current study demonstrated that, in relation to the control group, the AG and MG patients showed delays in average movement time of 92 ms and 70 ms, respectively. In addition, a previous comparative study of eye-hand coordination efficiency showed that patients with moderate-to-advanced stages of glaucoma committed more errors, and took longer to complete a linear tracking and aiming task compared to a normal-sighted control group [11].

In our study, the poorer performance of glaucoma patients in the complex visuomotor reaction task may be explained by a decrease in oculomotor efficiency that affects their visual search strategies when performing such tasks [37]. Glaucomatous visual field loss has been shown to affect eye movement patterns during visual search tasks mainly by reducing saccade rate [38]. Moreover, it has been noted that, compared to healthy subjects, during functional visuomotor tasks, patients with glaucoma have significantly slower and less accurate saccades [39]. Other studies have found that glaucoma patients demonstrate significantly different eye movement patterns due to aspects such as delayed fixations [40], reduced fixation rates and longer fixation durations [41]. Decreases in oculomotor function in patients with glaucoma may explain the poorer performance in the signal detection task by the glaucoma patient groups in our study. However, uncertainty remains as to exactly how glaucoma affects eye movement strategies [39]. In our opinion, the wide variation in glaucoma severity makes it impossible to identify one specific disorder in patients’ eye movements.

Alternatively, previous studies have reported that patients with glaucoma exhibited greater cognitive workload compared to controls, especially under strenuous visuomotor demand in dual task conditions requiring divided and selective attention [13,40,42], as well as in tasks where demands are placed on verbal memory and verbal working memory [43]. Experimental study results have shown that an increase in cognitive demand disproportionately worsened functional visual field performance in glaucoma patients compared to healthy controls during simulator driving tasks of increasing difficulty [42]. Similarly, in our study, the more complex task significantly differentiated between glaucoma patients and the control group.

It has been proposed that glaucoma, similar to other neurodegenerative diseases, affects cognition through similar biological pathways [43,44]. Degeneration of retinal ganglion cells and their axons in glaucoma also occurs in patients with Alzheimer’s disease [45]; both of these age-related neurodegenerative diseases may coexist in the elderly [46]. Lin et al. [47] demonstrated, over a period of 8 years, that patients with open-angle glaucoma were at a higher risk of developing of Alzheimer’s disease. Moreover, it has been observed that people with age-related macular degeneration, Fuchs’ corneal dystrophy, and glaucoma had lower cognitive scores than controls [48]. However, the pathogenic mechanisms linking retinal alterations to brain alterations in neurodegenerative diseases are still not completely known [44].

Importantly, despite the emerging trend of differences in test results (Table 2), we found no difference in visuomotor reaction task performance between individuals with AG and those with MG. Thus, moderate stage glaucoma may cause visuomotor impairment in older adults. Nonetheless, it is possible that, in real life, patients who have had a visual impairment for a longer duration may use compensatory mechanisms to improve the efficiency of their visual field scanning (e.g., modified eye and head movements) [19,20,49]. These compensatory mechanisms may explain the lack of differences in visuomotor task performance between moderate and advanced stage glaucoma patients in our study.

In glaucoma patients, this study found correlations between both age and visuomotor reaction test performance, and binocular visual field defect scores and visuomotor reaction test performance. Visuomotor skills play an important role in the performance of daily activities that are critical for an active and independent life. Aging is associated with reduced perceptual-cognitive ability in relation to visual tracking, perceiving moving stimuli, spatial location of stimuli in the visual field, and oculomotor function in visual search tasks [21,50]. Our findings demonstrate that this decline in glaucoma patients is associated with increasing age and worsening binocular glaucomatous visual field defect. Recent findings demonstrated that in glaucoma patients, binocular visual field capabilities appear to significantly affect motor performance (e.g., navigating and avoiding obstacles when walking) [51,52]. However, binocular visual field defects do not always lead to poorer performance, e.g., during a special-offer supermarket search task [49] and during driving [53].

Daily PA appeared to have a positive influence on visuomotor processing in patients with glaucoma. Four out of six associations between daily PA and visuomotor test performance were significant (Table 3). Specifically, PA levels accounted for 43% of the variance in complex reaction time. Many experimental studies have reported benefits of PA and exercise on cognitive function in older adults [54,55,56]. Regarding glaucoma, it has been suggested that low to moderate intensity aerobic exercise may reduce IOP and improve neural activity in the visual pathway [25,26,27]. However, heavy strength exercise could have a negative impact on IOP control [57], thus, highlighting the need for the careful design and implementation of PA programs for glaucoma patients. Moreover, when PA becomes difficult because of vision impairment, patients with glaucoma may limit their daily PA [58]. However, in the current study, we found no statistical differences in weekly energy expenditure related to PA between glaucoma patients and the control group.

The above findings should be interpreted with regard to some study limitations. Despite the considerable total number of participants (60 subjects), the number of participants in each group was relatively small. However, the participant exclusion criteria and comprehensive eye testing procedures used allowed us to obtain a more homogenous sample. Further, to the sample size, we assessed participants’ PA levels with a self-report questionnaire. Such questionnaires can result in inflated estimates of activity [59]; thus, caution is warranted when interpreting and applying the findings related to PA. Using alternative tools for physical activity assessment in free-living conditions (e.g., wearable accelerometer-based activity monitors) may increase the objectivity of PA measurement in future studies. Finally, physical fitness of older adults has been shown to influence activities of daily living; therefore, testing of physical performance should be considered as an inclusion criterion in future studies.

## 5. Conclusions

This study highlights the negative consequences of glaucoma on visuomotor processing in older adults, with a greater deterioration of executive function observed when undertaking a complex visuomotor reaction task. Despite an emerging trend, no difference in visuomotor reaction task performance was observed between individuals with advanced stage glaucoma compared to those with moderate stage glaucoma. Furthermore, the visuomotor decline in glaucoma groups was dependent on patient age, binocular visual field defects, and, in particular, PA levels. These findings may have significant implications for activities of daily living in older adults with glaucoma. Specifically, lower PA levels were associated with reduced visuomotor efficiency. These findings support PA programs for the maintenance or improvement of visuomotor functioning in older adults with glaucoma. For this purpose, prospective studies are needed to create evidence-based guidelines for PA recommendations in patients with glaucoma. Thus, a future study will be focused on finding the most effective methods of intervention among PA programs based on visuomotor training, aerobic exercise, and a combination of both, that will have a positive impact on the functional activities and quality of life in glaucoma sufferers.

## Figures and Tables

**Table 1:** Demographic and clinical characteristics of study participants.

Parameters	Advanced Glaucoma (*n* = 20)	Moderate Glaucoma (*n* = 20)	Controls (*n* = 20)	*p*-Value
	Mean ± *SD*	Mean ± *SD*	Mean ± *SD*	
Age (years)	66.35 ± 6.35	65.30 ± 7.50	64.55 ± 6.9	0.878
Women	9	11	8	0.626 ^#^
Men	11	9	12	
Height (cm)	169.10 ± 7.67	170.05 ± 8.80	172.55 ± 8.13	0.437
Weight (kg)	79.83 ± 9.94	80.18 ± 14.21	81.46 ± 8.85	0.754
PA (MET, min/week)	4580.00 ± 3432.07	5913.98 ± 4442.51	7588.78 ± 4554.29	0.077
Snellen BCVA				
Better eye	0.8 ± 0.2	0.9 ± 0.1 ^b^	1.0 ± 0.1 ^ccc^	0.001
Worse eye	0.6 ± 0.3	0.7 ± 0.2 ^bbb^	1.0 ± 0.1 ^ccc^	<0.001
Monocular VF				
MD better eye (dB)	−6.94 ± 5.65	−4.68 ± 2.75 ^bbb^	0.87 ± 0.78 ^ccc^	<0.001
MD worse eye (dB)	−21.45 ± 6.84 ^aa^	−9.42 ± 1.86 ^bbb^	0.37 ± 0.83 ^ccc^	<0.001
Binocular VF				
Defect scores (*n*)	34.15 ± 14.89 ^a^	14.70 ± 12.36 ^bb^	0.70 ± 1.13 ^ccc^	<0.001
Esterman coefficient score (%)	71.54 ± 12.41 ^a^	87.75 ± 10.30 ^bb^	99.42 ± 0.94 ^ccc^	<0.001

BCVA = best corrected visual acuity, MD = mean deviation, SD = standard deviation, VF = visual field, *^#^* the chi-square statistic. Pairwise comparisons: AG vs. MG ^a^ *p* < 0.05; ^aa^ *p* < 0.01; MG vs. CG ^b^ *p* < 0.05; ^bb^ *p* < 0.01; ^bbb^ *p* < 0.001; AG vs. CG ^ccc^ *p* < 0.001.

**Table 2:** Analysis of variance and pairwise comparisons for patients with glaucoma and healthy-sighted control group.

Parameters	Advanced Glaucoma	Moderate Glaucoma	Controls	*F*	*p*	ηp^2^
Simple visuomotor reaction test						
Simple reaction time (ms)	330.30 ± 54.35	323.67 ± 51.07	301.60 ± 51.63	2.261	0.113	0.074
Simple motor time (ms)	268.15 ± 85.11	248.65 ± 61.23 ^b^	191.60 ± 65.40 ^cc^	6.288	0.003	0.183
Total reaction time (ms)	598.45 ± 106.73	572.15 ± 92.56 ^b^	481.30 ± 105.85 ^cc^	7.275	0.002	0.203
Complex visuomotor reaction test						
Correct reactions (*n*)	43.65 ± 6.36	44.75 ± 6.59 ^b^	50.05 ± 4.39 ^cc^	6.716	0.002	0.193
Missed reactions (*n*)	14.55 ± 6.16	13.60 ± 6.31 ^b^	8.90 ± 4.81 ^cc^	5.515	0.001	0.165
Complex reaction time (ms)	1624.33 ± 257.20	1502.35 ± 275.52 ^bb^	1210.20±241.26 ^ccc^	14.427	<0.000	0.340

Pairwise comparisons: MG vs. CG ^b^ *p* < 0.05; ^bb^ *p* < 0.01; AG vs. CG ^cc^ *p* < 0.01; ^ccc^ *p* < 0.001.

**Table 3:** Pearson correlations of visuomotor reaction parameters, age, BCVA, VFDS and PA in glaucoma patients.

Parameters	Age	BCVA Better Eye	BCVA Worse Eye	MD Better Eye	MD Worse Eye	VFDS	PA
Simple visuomotor reaction test							
Simple reaction time	0.146	−0.130	0.040	0.256	0.063	0.284	−0.274
Simple motor time	0.396 *	−0.148	0.074	−0.223	−0.148	0.433 *	−0.330 *
Total reaction time	0.370 *	−0.200	0.076	−0.031	−0.143	0.470 *	−0.389 *
Complex visuomotor reaction test							
Correct reactions	−0.389 *	0.241	0.063	−0.057	0.230	−0.285	0.341 *
Missed reactions	0.335 *	−0.281	−0.048	0.084	−0.227	0.271	−0.308
Complex reaction time	0.650 **	−0.126	−0.076	0.039	−0.210	0.409 *	−0.659 **

* *p* < 0.05, ** *p* < 0.01, BCVA = best corrected visual acuity, MD = mean deviation, VFDS = binocular visual field defect scores, PA = physical activity.

## Data Availability

The data that support the findings of this study are available from the corresponding author, T.Z., upon reasonable request.
